# Adaptation of the “Osnabrück Scale for Therapy Adjustment and Identification of Psychological Complaints in Schizophrenia-OSSTI-TR” into Turkish culture and investigation of its psychometric characteristics

**DOI:** 10.3389/fpsyt.2026.1678728

**Published:** 2026-02-27

**Authors:** Elçin Babaoğlu, Yalçın Kanbay, Aydan Akkurt Yalçıntürk

**Affiliations:** 1 Uskudar University Health Sciences Faculty, Istanbul, Türkiye; 2 Artvin Coruh University Health Sciences Faculty, Artvin, Türkiye; 3 University of Health Sciences -Hamidiye Faculty of Nursing, Istanbul, Türkiye

**Keywords:** insight, reliability, schizophrenia, therapy adjustment, validity

## Abstract

**Aim:**

This study aims to adapt the “Osnabrück Scale for Therapy Adjustment and Identification of Psychological Complaints in Schizophrenia” (OSSTI) into Turkish and evaluate its psychometric properties. The study addresses the need for a practical self-report tool to simultaneously assess metacognitive awareness and treatment adherence in Turkish patients with schizophrenia.

**Methods:**

Data were collected from 335 patients diagnosed with schizophrenia. Following translation and back-translation, content validity was confirmed via expert panel. Construct validity was tested using Exploratory Factor Analysis (EFA) and Confirmatory Factor Analysis (CFA). Reliability was assessed through Cronbach’s alpha and split-half coefficients.

**Results:**

EFA identified a three-factor structure—Awareness, Denial, and Adjustment—explaining 58.03% of the total variance. While initial single-factor CFA was rejected, the final model demonstrated acceptable alignment with the theoretical framework. The scale’s overall internal consistency was Cronbach’s α = .73, with subscale coefficients ranging from .70 to .78. Convergent validity was supported by a significant moderate correlation with the Morisky Medication Adherence Scale (r = −.418, p<.001).

**Conclusion:**

The OSSTI-TR is a valid and reliable instrument for assessing therapy adjustment in schizophrenia. Its strong psychometric indices and metacognitive focus provide a robust framework for clinical evaluation and research in the Turkish context.

## Introduction

Schizophrenia is a relatively prevalent psychiatric disorder, with a lifetime prevalence of 0.48% (1–3). This disorder, which is associated with elevated therapy costs and significant functional impairment, typically manifests early adulthood and follows a chronic course (2). In order to facilitate adaptation to social life and improve quality of life, it is imperative that patients with this disorder adhere to their prescribed therapy and develop an understanding of their illness (3).

In the context of schizophrenia, non-adjustment to therapy has been demonstrated to result in an escalation of involuntary hospitalization, longer hospital stays, prolonged recovery from psychotic symptoms, a poor prognosis, and an increased risk of suicide (1–5). Research has indicated that the relapse rate among schizophrenia patients who do not adhere to regular antipsychotic medication therapy is five times higher than among those who do adhere to such therapy (3, 4). This problem of therapy non-adherence in patients also affects the people living with them, leading to burnout and increasing the cost of healthcare (1, 5, 6). Consequently, it is imperative to identify the factors that influence therapy adjustment in schizophrenia and to implement the necessary measures. Numerous factors have been identified as contributors to therapy adjustment, including patient and disease characteristics, the nature of the disease itself, the medications utilized, and environmental factors (7, 8).

Among patient-related factors, negative attitudes toward the illness and lack of insight prevent patients from developing healthy behaviors (1, 4, 5, 9). Insight in schizophrenia patients has been defined as the ability to recognize and accept the illness, be aware of its symptoms, and understand the need for therapy (10). Lack of insight is one of the main symptoms and problems of schizophrenia patients. Partial or complete lack of insight is seen in 80–85% of these patients (11). In schizophrenia, lack of insight is associated with higher relapse rates, increased hospitalization rates, longer hospital stays, a higher number of positive and negative symptoms, and suicide (4, 6, 12).

Insight into schizophrenia is not a fixed characteristic but a condition that can change over time depending on the natural progression of the disease or therapy (13). From a clinical perspective, insight is the capacity to recognize the presence of an illness, identify its symptoms, and accept the need for therapy by associating these symptoms with the illness (14). In patients with schizophrenia, insight also affects adjustment to therapy. Adjustment to therapy decreases relapses and improves quality of life in patients (15). Therefore, it is important for health professionals working in psychiatry to support patients with therapeutic interventions to improve insight and adherence to treatment. To plan these interventions, it is necessary to assess patients’ insight and adjustment to therapy.

The level of insight can be indirectly assessed by investigating how individuals diagnosed with schizophrenia perceive their symptoms, how they describe them, and what they expect from the progression of their illness and from their therapy. Various scales in the literature assess insight and therapy adjustment in patients with schizophrenia separately (16, 17). However, it can be challenging for patients with schizophrenia, who often have short attention spans and reduced cognitive functioning, to complete these scales. Therefore, new scales that assess both insight and therapy adjustment in patients with schizophrenia are needed. Healthcare professionals working with patients with schizophrenia should be able to improve these areas through relevant psychotherapeutic interventions, based on an assessment of patients’ insight and therapy adjustment. In this context, there is a need for short, easy-to-understand measurement tools that patients with schizophrenia can easily complete.

Metacognition is a series of cognitive activities that involve controlling, monitoring, and regulating cognitive processes, thereby directing and guiding behavior. Assessing metacognitive capabilities in individuals with schizophrenia is an important part of understanding the disease and developing effective therapy plans through research. Clinicians and researchers should be aware of the possibilities for accurately and reliably measuring areas of metacognition in order to address specific therapy options and monitor improvements in therapy or the progression of illness-related impairments (18). Traditionally, “” in schizophrenia patients has been viewed as a behavioral problem. However, according to the metacognitive model, the underlying problem of this non-adherence is cognitive. This requires clinicians to focus not only on prescribing medication but also on strengthening the patient’s ability to make sense of things.

To meet this need, Krupa (19) developed the Osnabrück Scale for and Identification of Psychological Complaints in Schizophrenia (OSSTI) in Germany, which assesses insight and therapy adjustment in patients with schizophrenia (original German title: Osnabrücker Skala zu Therapieeinstellung und Identifkation psychischer Beschwerden bei Schizophrenie). Waldorf (20), from the same research group, conducted validity and reliability studies of the OSSTI with 85 patients with schizophrenia at the University of Osnabrück (20). In this study, some items were removed, and the scale was reduced to ten items. The Cronbach’s α value for the OSSTI was found to be 0.79, and the average inter-item correlation was 0.28. The OSSTI is a self-report scale that considers insight and therapy adjustment together in patients with schizophrenia, making it advantageous for clinical assessment. Following the assessments, psychotherapeutic interventions to increase insight and adjustment to therapy can be planned and their effectiveness can be objectively evaluated. In Turkey, however, there is a notable lack of a comprehensive measurement tool that simultaneously assesses insight and therapy adjustment in schizophrenia patients in a practical, self-report-based manner. Most existing scales focus either solely on insight or solely on behavioral compliance, failing to address the patient’s motivation to participate in therapy within a metacognitive framework. The adaptation of the OSSTI-TR to Turkish culture aims to fill this psychometric gap between the insight levels and therapy intentions of schizophrenia patients.

The objective of this study is to establish the psychometric properties of the OSSTI and to undertake the OSSTI-TR investigation to determine its validity and reliability in Turkish.

Low metacognition in schizophrenia is associated with more severe symptoms and poorer functioning (18). Using the “Awareness” and “Adjustment” subscales of the OSSTI-TR as a measure of metacognition is thought to help predict not only symptom severity but also the patient’s long-term recovery potential more reliably. At this point, if the OSSTI-TR’s and insight dimensions are considered a reflection of metacognition, the scale can be thought of as an indirect and practical tool for monitoring the effectiveness of these specific therapies. Semi-structured comprehensive metacognitive interviews are time-consuming and require expertise. Within the scope of this study, the original 6-point response scale was reduced to a 3-point Likert scale (Agree, Undecided, Disagree) based on expert opinions and pilot study results. The main empirical rationale for this change is the limitations in cognitive functions, difficulties in focusing, and reduced abstraction abilities of the target population, individuals diagnosed with schizophrenia. The literature indicates that multiple-choice structures can lead to response confusion and undermine the reliability of measurement in groups experiencing cognitive difficulties (18, 21). Although simplifying the response options carries a theoretical risk of narrowing the score variance, it has improved data quality and the clinical applicability of the scale by enabling patients to distinguish between questions more clearly.

Furthermore, this study, conducted in the Turkish context, provides clinicians with fast and reliable data to predict patients’ post-hospital care processes. Using a shorter and more focused scale such as the OSSTI-TR to indirectly measure metacognitive capacity provides practicality in clinical routine and large-scale research. In this context, the OSSTI-TR is considered to be a cognitive tool that indirectly assesses the patient’s complex inner world and coping strategies with illness, i.e., metacognitive functioning, rather than a simple compliance/insight control measurement tool.

## Methods

### Aim of the study

This methodological study aims to determine the validity and reliability of “Osnabrück Scale for and Identification of Psychological Complaints in Schizophrenia-OSSTI-TR” in Turkish culture, which was developed to measure the therapy adjustment and psychological complaints of schizophrenia patients.

### Participants

The population of this study consists of individuals aged 18 years and older who are receiving inpatient or outpatient therapy for schizophrenia. The individuals in the sample were selected from among schizophrenia patients receiving inpatient treatment at a psychiatric hospital who were willing to participate in the study. Individuals diagnosed with schizophrenia receiving outpatient treatment were selected from among patients followed up at the polyclinic. Since this study is a scale adaptation study, factor analysis techniques must be used. The literature indicates that a sample size of 300 is considered adequate for factor analysis (22, 23). A total of 335 schizophrenia patients were reached during the data collection phase. Individuals who were over 18 years of age, voluntary, able to read and write, and receiving outpatient or inpatient therapy were included in the study, while patients in the acute phase and those who had been admitted to the clinic for less than 48 hours were excluded from the study.

### Data collection tools

In this study, the “Individual Information Form”, “Osnabrück Scale Draft Form for and Identification of Psychological Complaints in Schizophrenia-OSSTI-TR” and “Morisky Therapy adjustment Scale” were used as data collection tools.

#### Individual information form

The individual information form created by researchers contains a total of nine questions aimed at determining the age, gender, education and employment status, number of children, and other descriptive information of individuals diagnosed with schizophrenia, as well as characteristics related to their illness, such as type of therapy, date of diagnosis, year of first hospitalization, total length of hospitalization, and other characteristics. The information regarding the characteristics of the illness included in this form was obtained from the patient’s file/medical records.

#### OSSTI-TR draft form

A review of the literature revealed that Waldorf (20) developed a measurement tool for determining therapy adjustment and identifying psychological complaints in patients with schizophrenia. The original name of the scale, which has undergone validity and reliability studies in German, is “Osnabrücker Skala zu Therapieeinstellung und Identifkation psychischer Beschwerden bei Schizophrenie-OSSTI” (20). The OSSTI is a single-dimensional scale consisting of 10 items. The validity and reliability of this scale, which was tested on 85 schizophrenia patients, showed a Cronbach’s α value of 0.79 and an average inter-item correlation of 0.28. The scale has a 6-point Likert (0–6) structure, with 3 reverse-coded items (02, 09, 10). In the scale, “Strongly Disagree” = 0, “Somewhat Disagree” = 1, “Disagree” = 2, “Agree” = 3, “Strongly Agree”= 4 and “Strongly Agree” = 5, resulting in a score ranging from 0 to 50. On a scale with no cut-off point, as the score increases, the patient’s insight and compliance with therapy also increase (20). The name of the scale has been translated into Turkish as “Osnabrück Scale for and Identification of Psychological Complaints in Schizophrenia-OSSTI-TR”.

The translation-back translation method was applied to ensure the linguistic equivalence of the scale. First, the scale was translated into Turkish by two experts proficient in both languages; then, these forms were back-translated into the original language to check for any shifts in meaning. Following linguistic validity, a CVI was calculated by a panel of 10 experts, and the suitability of the scale items for Turkish culture and the clinical population was confirmed.

##### Translation of the OSSTI into Turkish

The translation of the OSSTI into Turkish was carried out by one expert translator in the field of German language and literature and one expert in the field of health. The scale items translated by the translators were reviewed, compared with each other in terms of suitability, and the necessary corrections were made.

##### Back-translation method

It is recommended that the back-translation be performed by experts who did not participate in the initial translation (24). Therefore, the back-translation was performed by two experts in the German language who did not participate in the initial translation, and the back-translations were then compared with the original scale. The original form obtained after back-translation was sent back to the author who developed the OSSTI scale for review to determine if there were any changes in meaning, and the translation was deemed appropriate by the author.

##### Experts’ opinion

Identifying insufficient concepts in the translation and detecting inconsistencies between the two languages with advanced translations requires expert opinion after translation (25). 10 Experts have stated that individuals diagnosed with schizophrenia are likely to have difficulty responding to items due to both the nature of the disease and the reduction in cognitive functioning caused by psychotropic therapies, and therefore a 3-point Likert scale is easier to understand than a 6-point scale. In line with expert opinions, the 6-point Likert scale was converted to a 3-point Likert scale. Thus, each item is answered as “I agree,” “I am undecided,” or “I disagree.” In line with expert opinions, the Content Validity Ratio (CVR) and Content Validity Index (CVI) values of the scale were calculated (26–28). The translated and linguistically validated draft form was sent to 10 experts consisting of psychiatrists, psychiatric nurses, and psychologists working in the field of psychiatry, and the CVR values of the form were calculated based on the expert assessments (29, 30).

In line with expert opinions, it was considered that the original 6-point Likert scale was not suitable for schizophrenia patients and Turkish culture, and a 3-point Likert scale was preferred for the adaptation of the scale to Turkish culture, resulting in the creation of the OSSTI-TR draft form. The OSSTI-TR draft form consists of a total of 10 items. Each item on the form is scored as “I disagree” = 0, “I am undecided” = 1, “I agree” = 2. Items 14, 15, and 16 are reverse scored. The scores that can be obtained from the scale range from 0 to 18. As the score on the scale increases, the individual’s adjustment to therapy and ability to recognize their psychological complaints increases.

#### The Morisky medication adherence scale

The scale developed by Morisky et al. (31) consists of four questions and closed-ended responses (yes/no). The validity and reliability of the scale in Turkish were established by Bahar and colleagues (32) with patients with bipolar mood disorder. If patients answer all questions with “no,” medication adherence is considered high; if one or two questions are answered with “yes,” medication adherence is considered moderate; and if three or four questions are answered with “yes,” medication adherence is considered low (32).

### Data collection

Data were collected from patients who agreed to participate in the study by having them fill out self-report forms. Data was collected electronically from schizophrenia patients participating in outpatient therapy services via Google Forms after obtaining ethics committee approval. Researchers prepared the OSSTI-TR questionnaire as a Google form and sent it to patients receiving therapy at the outpatient clinic via WhatsApp. Patients were asked to complete the questionnaire in their current environment. Patients were given access to the Google form only once to complete the scale. For patients diagnosed with schizophrenia who were receiving inpatient treatment at OSSTI-TR, researchers implemented the scale in face-to-face interviews at the clinic where the patients were receiving treatment.

### Data analysis

SPSS 26 and AMOS 23 software packages were used in the data analysis phase. In the analysis, descriptive statistics such as frequency, mean, and percentage values were provided, while correlation analysis was used to identify relationships. The construct validity of the scale was assessed using Exploratory Factor Analysis (EFA) and Confirmatory Factor Analysis (CFA). The reliability of the scale was tested using Cronbach’s alpha coefficient and split-half reliability.

### Ethical considerations

The OSSTI scale was adapted to Turkish culture with permission from the author who developed the measurement tool, which was obtained via email. An ethical approach was adopted throughout the study. Ethical approval was obtained from the Uskudar University Ethics Committee (61351342/020-270). Following ethical approval, a meeting was held with each participant to provide verbal information on ethical considerations, the voluntary nature of participation, what participation entails, and that participants may withdraw from the study at any time, with their personal information being removed from materials and publications. Verbal and written informed consent was obtained from all participants prior to the interviews. Written and verbal permission has been obtained from the legal guardian of schizophrenia patients who have a guardian. All procedures carried out in the study involving participants were in accordance with ethical standards and the Declaration of Helsinki. Participant information was kept confidential. Data were recorded by the researcher on a computer hard drive and stored in accordance with Institutional Ethical Review Boards (IRBs) guidelines.

## Results

The sample of this study consisted of 335 individuals aged 18 years and older who had been diagnosed with schizophrenia and were either hospitalized or receiving outpatient treatment. Of the sample, 26.9% were female, 24.6% were married, 45.4% were college graduates, and 50.7% were employed in a job that provided income. The mean age of the sample was 39.4 ± 9.8 (min: 18, max: 66). The mean number of hospitalizations for schizophrenia treatment among participants was 2.3 ± 1.1 (min: 1, max: 6) (see **Table 1**).

**Table 1:** Content validity values.

No	Nu	N	CVR (Nu-N/2)/(N/2)	Critic values	No	Nu	N	CVR (Nu-N/2)/(N/2)	Critic values
i1	9	10	0,80	.62	**i**	10	10	1,00	.62
i2	9	10	0,80	.62	**i7**	9	10	0,80	.62
i3	10	10	1,00	.62	**i8**	9	10	0,80	.62
i4	10	10	1,00	.62	**i9**	9	10	0,80	.62
i5	10	10	1,00	.62	**i10**	10	10	1,00	.62
Number of experts=10									
Critical value=.62									
CVI=.900									

*Items that were excluded from the study because they were deemed “Not Appropriate” or “Needs Revision” by experts and therefore did not meet the validity criteria; Nu, Number of experts who gave a “Required” opinion on the item; N, Number of experts who gave an opinion on the item; CVR, Content Validity Ratio, CVI, Content Validity Index.

### Validity of the OSSTI-TR

In this study, the views of 10 experts were taken for content validity. The critical value calculated for 10 experts is.62. The CVRs of the items on the scale ranged from .80 to 1. The CVI value calculated for the form was .900, and the CVI value was found to be greater than the CVR value (CVI>CVR).

#### Structural validity

**Figure 1:**
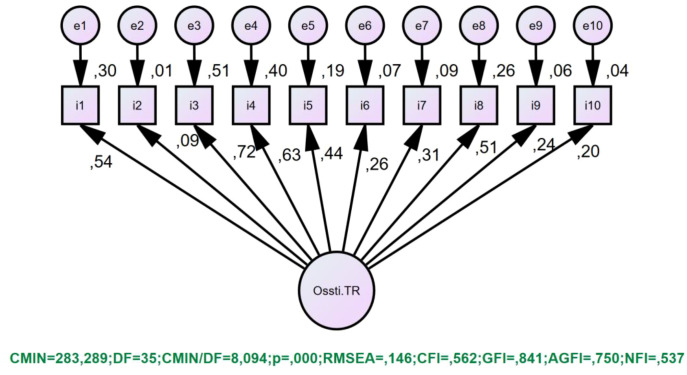
OSSTI-TR single-factor structure.

The structural validity of the OSSTI-TR was tested using first-order unidimensional DFA. When examining the fit indices for the structure, the following values were obtained: x2/df: 8.094, RMSEA: .146, CFI: .56, GFI: .84, AGFI: .75, and NFI: .54. The single-factor structure of the scale was rejected (see **Figure 1**).

Since the single-factor structure of the scale was rejected, an EFA was conducted to establish a new structure for the scale. The EFA was performed on 10 items using maximum likelihood, principal component analysis, and Direct Oblimin rotation with seven iterations. The adequacy of the sample for CFA was evaluated using the Kaiser-Meyer-Olkin and Bartlett tests. The Kaiser-Meyer-Olkin test yielded a value of 0.763, and the Bartlett tests were also conducted. According to this evaluation, the relationship was found to be significant (χ2 = 507.030; df=36; p<0.001). At this step, a three-factor structure with eigenvalues greater than 1 was obtained. When the factor loadings of the items were examined, it was observed that the factor loadings ranged from .632 to .795. Item i8 was removed from the scale because its factor loading was not at the desired level and it showed high cross-loadings with multiple factors. These three factors were determined as “Awareness/Denial/Adjustment factors,” as in the German version of the OSSTI. The total variance explained by these three factors was found to be 58.03%. When the variance explained by each factor was examined individually, the Awareness factor accounted for 26.4%, the Denial factor for 20.3%, and the Adjustment factor for 11.3% (see **Table 2**).

**Table 2:** Factor structure and explained variance ratio of the OSSTI-TR.

No	Items	F1	F2	F3
**i1**	After I am discharged from the hospital, I will continue to need medical (psychiatric) or therapeutic care.	.791		
**i3**	I need to receive therapy/live in the hospital to cope with my psychological problems.	.776		
**i4**	I have early warning signs of psychological problems.	.686		
**i9**	I will not do anything about my psychological problems because they will go away on their own.		.795	
**i2**	I am healthy and have no psychological problems.		.780	
**i10**	I can stay healthy without medication.		.659	
**i6**	I will tell my family, friends, or relatives about my psychological problems to prevent misunderstandings in the future.			.710
**i5**	I will closely follow my doctor’s medical advice.			.698
**i7**	I will seek professional help (e.g., psychiatrist, other specialist, therapist) if I have psychological problems in the future.			.632
	**Explained variance:**	**26.4**	**20.3**	**11.3**
	**Total variance:**	**58.03**		
	**Kaiser-Meyer-Olkin(KMO) (.763) and Bartlett’s Test (x2=507.030, df=36, p<.001)** Principal Components Analysis was used for extraction, and Direct Oblimin (7 iterations)was used for rotation			

**F1,** Awareness; **F2,** Denial; **F3,** Adjustment.

#### Internal validity

The internal validity of the items comprising the scale was tested by comparing the upper and lower groups (27%). It was determined that there was a statistically significant difference between the mean scores of the upper group, which had high mean scores in both the factors and the total scale, and the lower group, which had low mean scores (p<.001) (see **Table 3**).

**Table 3:** 27% lower–upper group comparison.

Factor	Group	n	X	Sd.	t	p
F1. Awareness	Lower Group	90	.10	.30	−54.199	.000
	Upper Group		4.93	.79		
F2. Denial	Lower Group	90	.80	.60	−60.847	.000
	Upper Group		5.69	.47		
F3. Adjustment	Lower Group	90	1.07	.88	−32.419	.000
	Upper Group		5.16	.81		
OSSTI-TR	Lower Group	90	4.36	1.11	−37.521	.000
	Upper Group		13.60	2.05		

#### Convergent and known-groups validity

In order to examine the convergent validity of the scale, the relationship between the OSSTI-TR and the MMAS was examined. Pearson correlation analysis was used to determine the relationship between the scales. As a result of the convergent validity analysis, a negative and moderate relationship was found between the OSSTI-TR and the MMAS (r= –.418, p<.001).

According to the Known-Groups Validity results, no significant difference was found in the OSSTI-TR scores according to gender and education level variables (p>.05). On the other hand, a significant difference was found between the groups in terms of marital status and employment status. Married individuals had significantly higher the OSSTI-TR scores than single individuals (t: 2.832; p<.05). The same applies to unemployed individuals. The average OSSTI-TR scores of unemployed individuals were higher than those of the employed group (t: −4.331; p<.001) (see **Table 4**).

**Table 4:** Known-groups validity of the OSSTI.

Variable	Category	n	x	Sd.	T	P
Gender	Female	90	8.63	3.48	.143	.886
	Male	245	8.57	3.84		
Marital Status	Married	156	9.20	4.05	2.832	.005
	Single	179	8.05	3.36		
Level of education	High School	183	8.61	3.97	.115	.908
	Undergraduate	152	8.56	3.46		
Employment status	Working	170	7.74	3.18	−4.331	.000
	Not working	165	9.46	4.07		
		n	r	p		
	**Age**	335	.106	.053		
	**Number of hospitalizations**	335	.139	.011		

n, Sample size; x, Mean; Sd.: Standard deviation; t, t-test value; r, Pearson correlation coefficient; p, Statistical significance.

### Reliability of the OSSTI-TR

In order to test the reliability of the structure obtained in this study, Cronbach’s α reliability coefficient, Gutman’s coefficient, and Spearman-Brown two-half test reliability were calculated. As a result of the analyses, Spearman-Brown was found to be in the range of .619–.764. The Cronbach α value of the OSSTI-TR scale was calculated as .73. The Cronbach α values for the scale’s factor structures ranged from .70 to .78 (see **Table 5**).

**Table 5:** OSSTI-TR reliability of the OSSTI-TR.

Factor	r	Gutman	Cronbach α
F1. Awareness	.764	.70	.78
F2. Denial	.695	.68	.76
F3. Adjustment	.619	.67	.70
OSSTI-TR	.650	.67	.73

r, Spearman Brown Coefficient.

## Discussion

Adjustment to therapy and a patient’s ability to recognize their psychological issues are key to preventing episodes and repeated hospitalizations in patients with schizophrenia. One of the most important factors affecting therapy adjustment is patients’ lack of insight into their illness. This leads to hopelessness about the illness and non-compliance with medication. In clinical practice, a standardized approach to assessing both therapy adjustment and insight in patients with schizophrenia has yet to be established (33).

Validated tools for assessing these factors include the Positive and Negative Syndrome Scale (PANSS) for schizophrenia (34), the Three Components of Insight Assessment Scale (35), the Wisconsin Card Sorting Test (36), and the Medication Adherence Scale (MAS) (34). Reliability studies have been conducted on these tools and the results have been published in the national literature (18). Additionally, the “Insight Scale for Psychosis”, developed by Birchwood et al. (16), has been validated, found to be reliable, and is used in clinical practice. However, there is no Turkish validity or reliability study for this scale. With the exception of the PANSS, all of the other scales are self-report scales that must be completed by the patient. They are not specific to schizophrenia, but generally assess therapy adjustment and insight.

The OSSTI is a self-report scale developed by Krupa (19), based on the “Scale for Awareness of Mental Illness” (37) and Birchwood’s Insight Scale (16). This scale has the advantage of assessing both therapy adjustment and insight into the illness in patients with schizophrenia (19). The scale consists of three subscales containing 24 items in total, and is used as a six-point Likert scale. However, Krupa (19) was unable to obtain sufficient data on a large enough sample size to conduct a validity and reliability study, and was only able to study the construct validity of the scale with 26 patients (19). The Cronbach’s alpha value of the scale in this first version was found to be 0.76. Waldorf, from the same study group, conducted validity and reliability studies on the German version of the OSSTI at the University of Osnabrück with 85 patients with schizophrenia (20). In this study, some items were removed and the number of items on the scale was reduced to 10. The Cronbach’s alpha value for the OSSTI was found to be 0.79, with an average inter-item correlation of 0.28.

This study examined the validity and reliability of the OSSTI in a Turkish-speaking sample. The sample consisted of individuals aged 18 years and over who had been diagnosed with schizophrenia. Of the participants, 26.9% were female, 24.6% were married, 45.4% were college graduates and 50.7% were employed in a job that provided income. The mean age of the sample was 39.4 ± 9.8 years (range: 18–66 years), and the mean number of hospitalizations was 2.3 ± 1.1 (range: 1–6). The OSSTI-TR is the first assessment tool in the Turkish national literature to simultaneously assess therapy adjustment and psychological complaints in patients with schizophrenia. The OSSTI-TR uses a 3-item Likert scale and consists of a total of nine items. The Cronbach α value of the OSSTI-TR is .73, which is considered good, and it correlates with the MMAS scale (.418, p< 0.001). (r = –0.418, p< 0.001). Findings from validity and reliability studies indicate that the Turkish version of the OSSTI-TR is a valid and reliable measurement tool for patients with schizophrenia.

### Validity of the OSSTI-TR

Initially, this study examined the content validity findings of the OSSTI-Tr draft scale. Content validity was assessed using the Lawshe technique to calculate CVR and CVI. This technique provides a systematic approach to examining the content validity of scale items in scale development and validity studies, offering an objective evaluation based on expert opinion (27, 38). To this end, ten experts in the fields of psychiatry, psychology and psychiatric nursing were consulted, and the CVR of the items and the CVI of the scale as a whole were examined. In this study, the critical value calculated for the content validity of the ten experts was 0.62. The CVRs of the scale’s items ranged from 0.80 to 1.00, indicating that each item has sufficient validity. The CVI value calculated for the entire form was 0.900 and was found to be greater than the CVR value (CVI > CVR). These findings were interpreted as indicating that the 10-item form provides overall content validity (see **Table 1**).

Data obtained from a sample of 335 individuals were tested using CFA to verify the construct validity of the OSSTI-TR. For the analysis, goodness-of-fit indices and item-item reliability coefficients were examined. In the proposed model, Chi-square Goodness-of-Fit Test (χ2), Root Mean Square Error of Approximation (RMSEA), Goodness of Fit Index (GFI), Comparative Fit Index (CFI), Normative Fit Index (NFI) and Adjusted Goodness of Fit Index (AGFI) were used as fit indices (39, 40). The construct validity of the OSSTI-TR was tested using first-order single-factor CFA. It was found that the items contributed meaningfully to the structures they belonged to, but the factor loadings of some items were not at an acceptable level. When examining the fit indices for the structure, the following values were obtained: x²/df: 8.094, RMSEA: .146, CFI: .56, GFI: .84, AGFI: .75, and NFI: .54 (see **Figure 1**). Since the obtained goodness-of-fit values did not meet the minimum acceptable goodness-of-fit values recommended in the literature, the single-factor structure of the scale was rejected (41–43).

Since the single-factor structure of the scale was rejected, EFA was conducted to establish a new structure and reveal the scale’s structure. EFA is used when a researcher has insufficient evidence to form a hypothesis about the underlying factors of the data (44). Therefore, EFA identifies the number and type of factors in the desired scale. As a result of the EFA analysis, a minimum acceptable factor loading of 0.50 was accepted for each item (45). A prerequisite for exploratory factor analysis is the presence of correlation between variables. The Bartlett sphericity test is used to determine whether there is sufficient correlation among variables (46). Bartlett’s sphericity and Kaiser-Meyer-Levene tests were used to check the adequacy of the sample; Bartlett’s test showed a high level of significance, indicating a high-quality sample (χ² = 507.030; df = 36; p< 0.001). Since Bartlett’s sphericity test is sensitive to sample size, the Kaiser-Meyer-Alkin test (0.763) was also examined. An index value greater than 0.5 is considered acceptable; however, a value higher than 0.9 is more ideal (44). Eigenvalues and scree plots were used to determine the number of factors to be extracted (44). As a result, three sub-dimensions were identified in the scale. These sub-dimensions are Awareness, Denial, and Adjustment. The variance explained by the Awareness sub-dimension is 26.4%, the variance explained by the Denial sub-dimension is 20.3%, and the variance explained by the Adjustment sub-dimension is 11.3%. It was found that the three-factor structure of the scale accounts for 58.03% of the total variance, and it was determined that the items included in the structure contribute sufficiently to the scale (see **Table 3**).

The first version of OSTTI, developed by Krupa, was a 6-item Likert scale consisting of 3 subscales and a total of 24 items. The same scale was studied by Waldorf (20) with a larger sample size, and the number of items was reduced to 10. Waldorf (20) retained the 6-point Likert structure in the scale. The draft form of the OSSTI-TR was organized in a 3-point Likert structure in line with expert opinions. The reason for this was to maintain the comprehensibility of the scale due to the possibility of schizophrenia and the therapies applied reducing cognitive functioning. When examining the factor loadings of the items in the 10-item scale, it was observed that the factor loadings ranged from .632 to .795. Item 1_8_ (I have mental health problems and am receiving therapy here) was removed from the scale because its factor loading was not at the desired level and it had a high level of cross-loading on multiple factors (see **Table 2**). As a result of the analyses, the OSTTI-TR consists of a 3-item Likert scale with a total of 9 items and three subscales.

One of the subscales of the OSSTI-TR, the “Awareness” subscale, is calculated using items 1, 2, and 3. This subscale reflects the individual’s ability to recognize their mental symptoms, distinguish early warning signs of illness, and recognize the need for therapy. This subscale expresses the person’s insight into their own mental health status and acceptance of the need for treatment. In schizophrenia, insight is one of the critical determinants of therapy adjustment and long-term recovery. Disease awareness plays an important role in maintaining mental well-being by increasing motivation for treatment and adherence to treatment (37). The variance explained by this factor is 26.4%, and high scores on this factor indicate high therapy adjustment and awareness of psychological complaints in schizophrenia.

The “Denial” subscale refers to the individual’s denial of the existence of mental problems, belief that treatment is not needed, and unrealistic perceptions about their own well-being. This subscale reflects the lack of insight into illness and resistance to treatment commonly seen in schizophrenia. The denial mechanism can negatively affect treatment processes by preventing individuals from accepting their mental health problems and may lead to poor clinical outcomes (47). The items in this factor are reverse scored. This factor explains 20.3% of the variance, and high scores on this factor indicate low levels of therapy adjustment and denial of psychological complaints in schizophrenia. This subscale is measured by items 14, 15, and 16 of the scale, and scores are calculated inversely in this subscale (“Disagree” = 2, “Undecided” = 1, “Agree” = 0).

The “Adjustment” subscale covers the individual’s active participation in treatment processes, compliance with doctor’s recommendations, tendency to seek professional help, and behavior in utilizing social support resources. This dimension is critical in terms of increasing treatment adherence and long-term functionality in schizophrenia. Therapy adjustment is a fundamental factor in controlling symptoms, reducing hospitalization rates, and improving quality of life in chronic mental illnesses such as schizophrenia (21). This factor explains 11.3% of the variance, and high scores on this factor indicate high therapy adjustment and consistency in identifying psychological complaints in schizophrenia. This subscale is measured by items 17, 18, and 19 of the scale.

The internal validity of the scale’s items was examined through a comparison of the upper and lower 27% groups. The 27% method is a statistical technique that quantifies the extent to which an item differentiates between participants with high and low scores. Participants are then placed into two distinct groups based on their total scale scores: the top 27% are designated as the upper group, while the bottom 27% are classified as the lower group. A statistical analysis was conducted to compare the mean scores of the upper group, which exhibited elevated mean scores in both the factors and the total scale, and the lower group, which demonstrated low mean scores. The results indicated a statistically significant difference between these two groups (p<.001). The constructed structure revealed that the scale possesses the capacity to accurately differentiate between individuals exhibiting high or low levels of and psychological complaints, thereby demonstrating internal validity (see **Table 3**).

Convergent validity of the scale was examined by comparing the OSSTI-TR with the MMAS Scale. Since high scores on the MMAS scale reflect low therapy adjustment, a negative relationship was expected between OSSTI-TR and MMAS scores. A negative and moderate relationship was found between OSSTI-TR and the MMAS Scale (r= –.418, p<.001). This result shows that as OSSTI-TR scores increase, individuals’ also increases, whereas as scores on the MMAS scale increase, adjustment decreases. This result supports the convergent validity of the OSSTI-TR, as it demonstrates a meaningful relationship with a scale measuring similar concepts.

To examine the Known-Groups Validity of the scale, the OSSTI-TR was tested to see if it showed meaningful score differences between different groups. If the scale shows meaningful differences as expected (e.g., based on variables such as age, gender, marital status), this provides additional evidence that the scale accurately measures the conceptual structure. According to the Known-Groups Validity results, no meaningful difference was found in OSSTI-TR scores based on gender and education level variables (p >.05). However, a significant difference was found between the groups in terms of marital status and employment status. Married individuals had significantly higher OSSTI-TR scores than single individuals (t: 2.832; p<.05). The same was true for unemployed individuals. The mean OSSTI-TR scores of unemployed individuals were higher than those of the employed group (t: −4.331; p<.001). These findings show that single individuals and unemployed individuals have higher levels of and psychological complaints identification in schizophrenia. The study also examined the relationship between age, number of hospitalizations, and OSSTI-TR mean scores. No significant relationship was found between age and OSSTI-TR (r: .106; p>.05), while a statistically significant relationship was found between the number of hospitalizations and OSSTI-TR (r: .139; p<.05). According to this finding, and the level of identification of psychological complaints increase with the number of hospitalizations in schizophrenia (see **Table 4**).

### Reliability of the OSSTI-TR

Reliability is defined as the ability of test or scale results to accurately reveal the conceptually related phenomenon (48). To test the reliability of the structure in this study, Cronbach’s α reliability coefficient, Gutman’s coefficient, and Spearman-Brown two-half test consistency were calculated. Although a reliability coefficient above.70 is considered sufficient for a Likert-type scale, it should be as close to 1 as possible (46, 49). In the two-half test consistency, the correlation between the two halves is expected to be as high and meaningful as possible. According to the findings, moderate to high levels of correlation were observed between the two halves in the scale factors and the scale total (Spearman-Brown: .619–.764). The Cronbach α value for the two halves of the scale ranged from .70 to .78. The reliability coefficients obtained are within the limits recommended in the literature (46). When the two-half test reliability and Cronbach α reliability coefficient of the scale are evaluated together, the reliability of the three-factor structure obtained is considered to be at a sufficient level (see **Table 5**).

The Cronbach’s alpha value of the OSSTI-TR was found to be .73. The Cronbach’s alpha value of the German original version of the OSSTI, which was studied with a sample of 85 people, was .79. It can be seen that the Cronbach’s alpha values of the OSSTI and OSSTI-TR are close to each other. According to the literature, it is known that as the sample size increases in scale studies, the Cronbach’s alpha value becomes more reliable and stable. If the sample size is small, the Cronbach’s alpha value may be less accurate and more volatile, which may not accurately reflect the reliability of the population. Therefore, a sufficient sample size is generally important for accurately assessing the reliability of a scale. In the literature, a sample size of 300 is considered a good value for factor analysis (22, 23). The sample size in this study is 335. This suggests that the Cronbach Alpha value of the OSSTI-Tr scale is more reliable and stable. The scale’s 3-point Likert structure and moderate reliability coefficients have ensured that measurements are consistent with patients’ cognitive capacities during clinical application. This is a conscious methodological choice that enhances the clinical reliability and comprehensibility of the data obtained, beyond statistical variance.

It is widely acknowledged that recovery rates are low in schizophrenia patients, who often experience relapses and hospitalizations. Schizophrenia has a negative effect on patients’ quality of life due to its recurrent nature and the need for long-term pharmacological therapy. Sampogno et al. (50) reported that the effect of antipsychotic drugs on the quality of life of schizophrenia patients is closely related not only to symptom control but also to treatment adherence, social and cognitive functioning (50). Improving patients’ quality of life depends not only on the biological efficacy of the drugs but also on the patient’s motivation to use the medication, which is linked to their metacognitive capacity. High metacognitive capacity leads to high, which maximizes the potential of the drugs to improve quality of life (18, 50). In schizophrenia, “recovery” now means not only symptom remission but also high quality of life and social functioning. Improving insight strengthens the patient’s social and cognitive abilities through methods such as metacognitive therapy (18). When the “Awareness” and “Adjustment” subscales of the OSSTI-TR are considered as indirect indicators of metacognitive capacity, this measurement tool is thought to be clinically useful for monitoring patient-centered outcomes. By measuring and insight, the OSSTI-TR enables therapy teams to quickly identify cognitive barriers that may hinder the potential impact of medications on quality of life.

The strongest and most prominent predictor of rehospitalization is poor therapy adjustment. Particularly in First-Episode Psychosis, high rates of rehospitalization are commonly observed within 12 months. Patients who discontinue or interrupt medication during this period have a significantly higher risk of relapse (51). The “Adjustment” subscale of the OSSTI-TR allows for the objective assessment of the extent to which the patient adheres to therapy (medication and psychosocial) during the post-discharge period. High adjustment scores on the OSSTI-TR provide an important indicator that the risk of readmission will be low during the 12-month follow-up period. The OSSTI-TR provides a quick and practical assessment that shows increased non-compliance before readmission, helping clinicians provide supportive interventions before hospitalization is required. The **“**Awareness” subscale of the OSSTI-TR measures the patient’s ability to accept their illness and treatment needs, indirectly assessing the fundamental cognitive deficits that may hinder treatment participation and social integration. Therefore, it is considered that the OSSTI-TR is an effective tool for measuring the determinants of readmission in schizophrenia and psychosis in a practical manner, and for predicting and monitoring the risk of readmission and recovery in patients.

## Conclusion

Therapy adjustment and insight are important factors in maintaining the quality of life of patients with schizophrenia. Mental health professionals can increase and insight in schizophrenia patients through psychotherapeutic interventions. Objective measurement tools that assess and insight in schizophrenia patients are needed so that professionals can plan, implement, and evaluate interventions specific to their fields. Due to the short attention span of schizophrenia patients, self-report scales that assess and psychological complaints together are more advantageous at this point. However, there is no measurement tool in our country that assesses psychological complaints together in schizophrenia patients. This study is expected to address this need.

The OSSTI-TR is a measurement tool that can be used with both outpatients and inpatients. The OSSTI-TR is easy to use and score, practical, and can be administered in a short time, which are also considered advantages. This study presents the initial psychometric findings for the Turkish adaptation of the OSSTI-TR. The three-factor structure and moderate reliability coefficients (α = .73) indicate that the scale should be considered an initial adaptation. The low fit of the original single-factor model in CFA led to the emergence of a different factor structure in Turkish culture; this may reflect the effects of cultural/linguistic adaptation and the 3-point Likert format on cognitive processing. The findings indicate that the OSSTI-TR is a promising measurement tool for clinical applications, but it is not yet fully mature. Therefore, the scale needs to be tested with confirmatory analyses in different samples, and test-retest reliability, measurement invariance, and cross-cultural validation studies need to be conducted.

This study has some limitations. First, the newly constructed three-factor structure could not be validated with CFA in an independent sample, which constitutes a methodological limitation due to the difficulty of reaching schizophrenia patients. Second, although the reduction of response options from a 6-point to a 3-point Likert scale was based on clinical reasons (cognitive load, attention span, expert opinions), it is an adaptation that could affect measurement sensitivity. Third, although no significant differences were found in the analyses, the data were collected using both online and face-to-face methods, and the difference in methods may potentially introduce bias in the measurement. Finally, this study provides only initial validity and reliability evidence for the OSSTI-TR; the scale requires additional psychometric validation in longitudinal use, different cultural settings, and larger samples.

### Scale instructions

**Figure 2:**
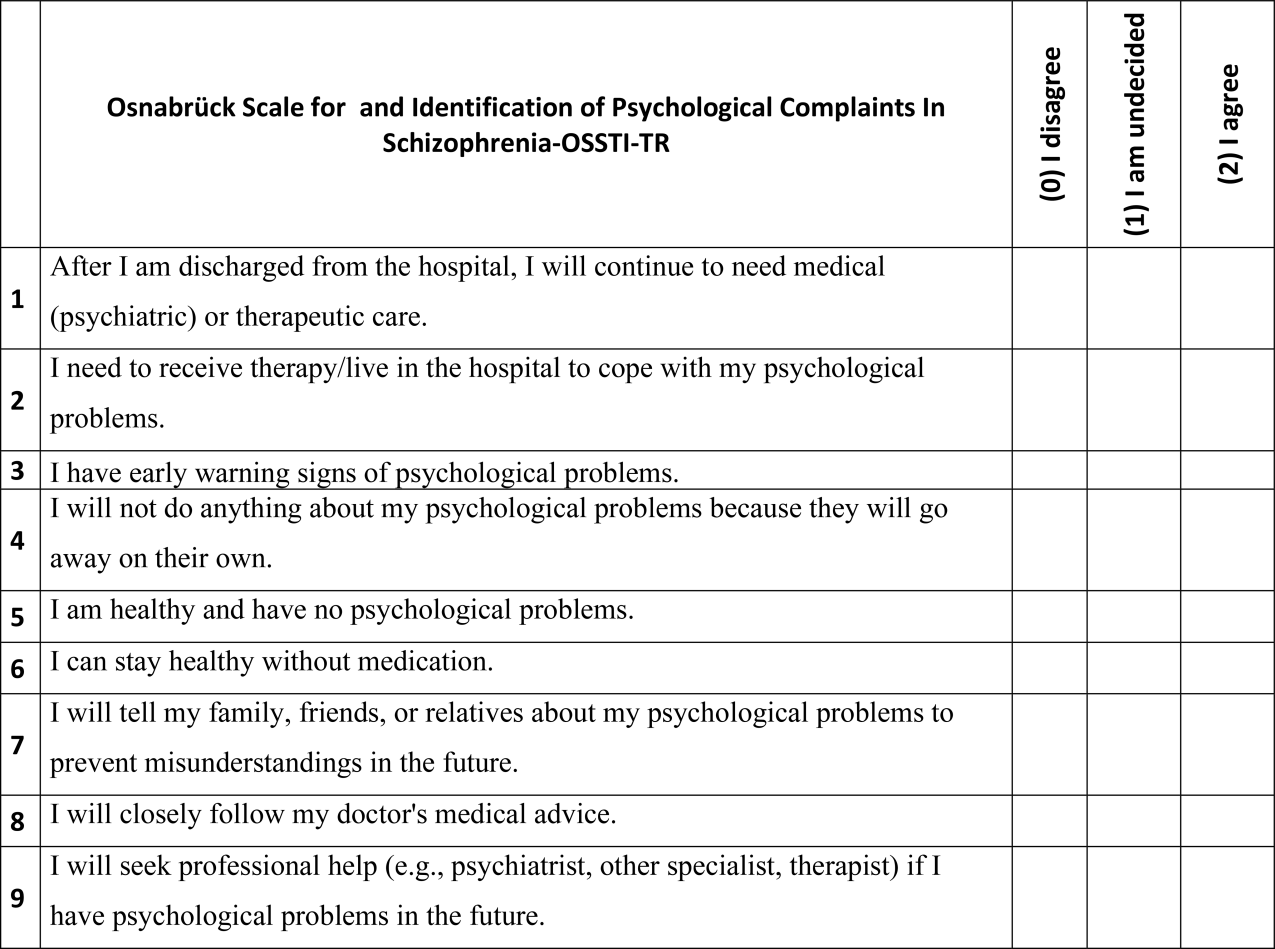
Osnabrück scale for and identification of psychological complaints in schizophrenia-OSSTI-TR.

The OSSTI-TR scale is a self-report measurement tool consisting of a total of 9 items in a 3-point Likert scale. The scale has 3 subscales: awareness/denial and adjustment (see **Figure 2**).

The awareness subscale (i_1_–i_3_) reflects the individual’s ability to recognize their psychological complaints, distinguish early warning signs of their illness, and recognize the need for therapy. This dimension expresses the person’s insight into their own mental health status and acceptance of the need for therapy. High scores on this factor indicate high and awareness of psychological complaints in schizophrenia.

The denial subscale (i_4_–i_6_) refers to the individual’s denial of the existence of psychological complaints, belief that they do not need treatment, and unrealistic perception of their own well-being. This dimension reflects the lack of insight into the illness and tendency to resist treatment commonly seen in schizophrenia. The items in this factor are reverse scored. High scores on this factor indicate low levels of and denial of psychological complaints in schizophrenia.

The adjustment subscale covers behaviors such as active participation in treatment processes, compliance with doctor’s recommendations, tendency to seek professional help, and use of social support resources. High scores on this factor indicate high and high agreement in identifying psychological complaints in schizophrenia.

The OSTII-TR is scored as “I disagree” = 0, “I am undecided” = 1, and “I agree” = 2. Three items on the scale are reverse-scored (i_4_–i_6_), and scores on the scale range from 0 to 18. As the score on the scale increases, the patient’s insight into schizophrenia and also increases. The scores that can be obtained on the original scale range from 0 to 50. In order to make consistent comparisons between samples in the studies, the OSSTI-TR has been standardized in the 0–50 score range.

To standardize scores obtained from the scale to a range of 0–50, the following formula should be used:


$$Standardized\;Score=\left(\frac{Original\;\;Score}{Maksimum\;Score}\right)\times 50$$

For example, the standardized score for a person with a raw score of 9 can be calculated as follows:


$$Standardized\;Score=\left(\frac{9}{18}\right)\times 50=25$$

In this case, a person who scored 9 on the scale has a standardized score of 25.

## Data Availability

The raw data supporting the conclusions of this article will be made available by the authors, without undue reservation.
